# Supplementary material for: A CRISPR-Cas12a-derived biosensing platform for the highly sensitive detection of diverse small molecules
Source: Nat Commun. 2019 Aug 14;10:3672. doi: 10.1038/s41467-019-11648-1 (PMC6694116; doi:10.1038/s41467-019-11648-1)
Supplement: Supplementary file 1 — Supplementary Information [file 41467_2019_11648_MOESM1_ESM.pdf]

Supplementary Information

A CRISPR/Cas12a-derived biosensing platform for the highly sensitive detection of diverse small molecules

Liang et al.

Supplementary Figures

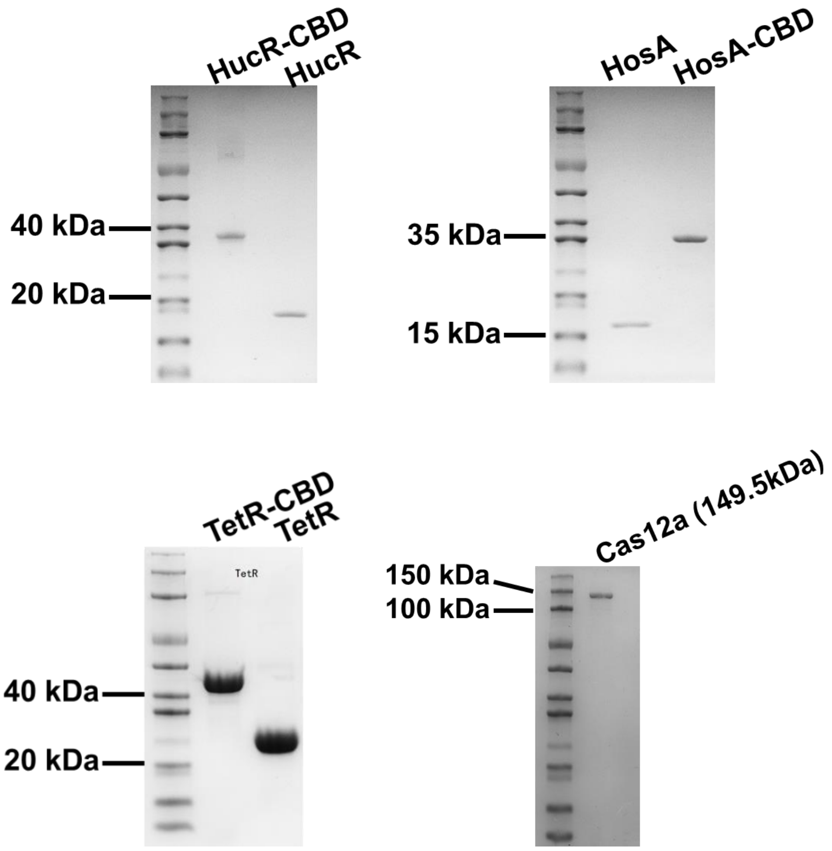

Supplementary Figure 1. Expression and purification of CBD-HucR, CBD-HosA, CBD-TetR and Cas12a.

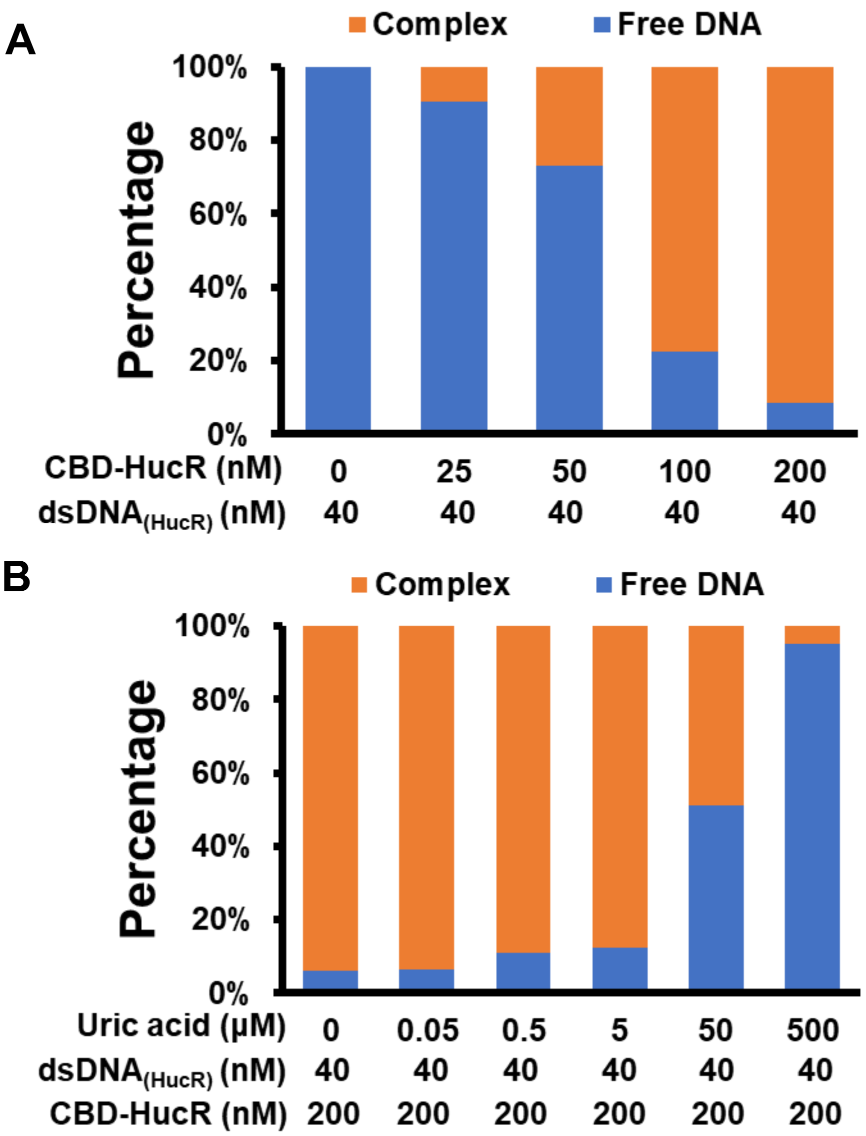

Supplementary Figure 2. Relatively quantitative analysis of EMSA data obtained by densitometric analysis of the dsDNA-CBD-HucR complex. **A.** EMSA with the CBD-HucR and dsDNA. The concentration of $dsDNA_{(HucR)}$ was 40 nM, CBD-HucR was added to the system with a final concentration of 0, 25, 50, 100, 200nM. **B.** Evaluation of CBD-HucR allosteric activity in the presence of uric acid by EMSA. The concentrations of $dsDNA_{(HucR)}$ and CBD-HucR was 40nM and 200nM, respectively. Uric acid was added to the system with a final concentration of 0, 0.05, 0.5, 5, 50 and 500μM. Densitometric analysis was performed by gel image software (BioRad Inc.).

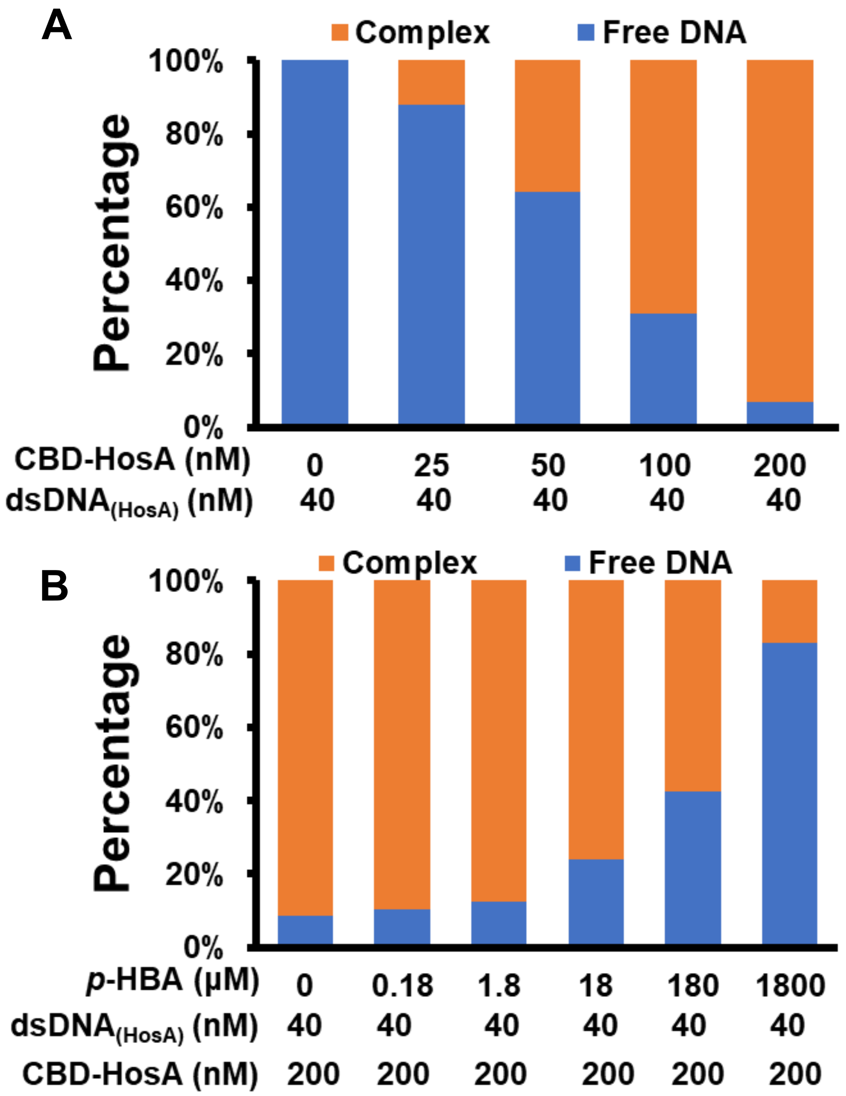

Supplementary Figure 3. Relatively quantitative analysis of EMSA data obtained by densitometric analysis of the dsDNA-CBD-HosA complex. **A.** EMSA with the CBD-HosA and dsDNA. The concentration of $dsDNA_{(HosA)}$ was 40 nM, CBD-HosA was added to the system with a final concentration of 0, 25, 50, 100, 200nM. **B.** Evaluation of CBD-HosA allosteric activity in the presence of uric acid by EMSA. The concentrations of $dsDNA_{(HosA)}$ and CBD-HucR was 40nM and 200nM, respectively. The concentrations of $dsDNA_{(HosA)}$ and CBD-HosA was 40nM and 200 nM, respectively. *p*-HBA was added to the system with a final concentration of 0, 0.18, 1.8, 18, 180 and 1800μM. Densitometric analysis was performed by gel image software (BioRad Inc.).

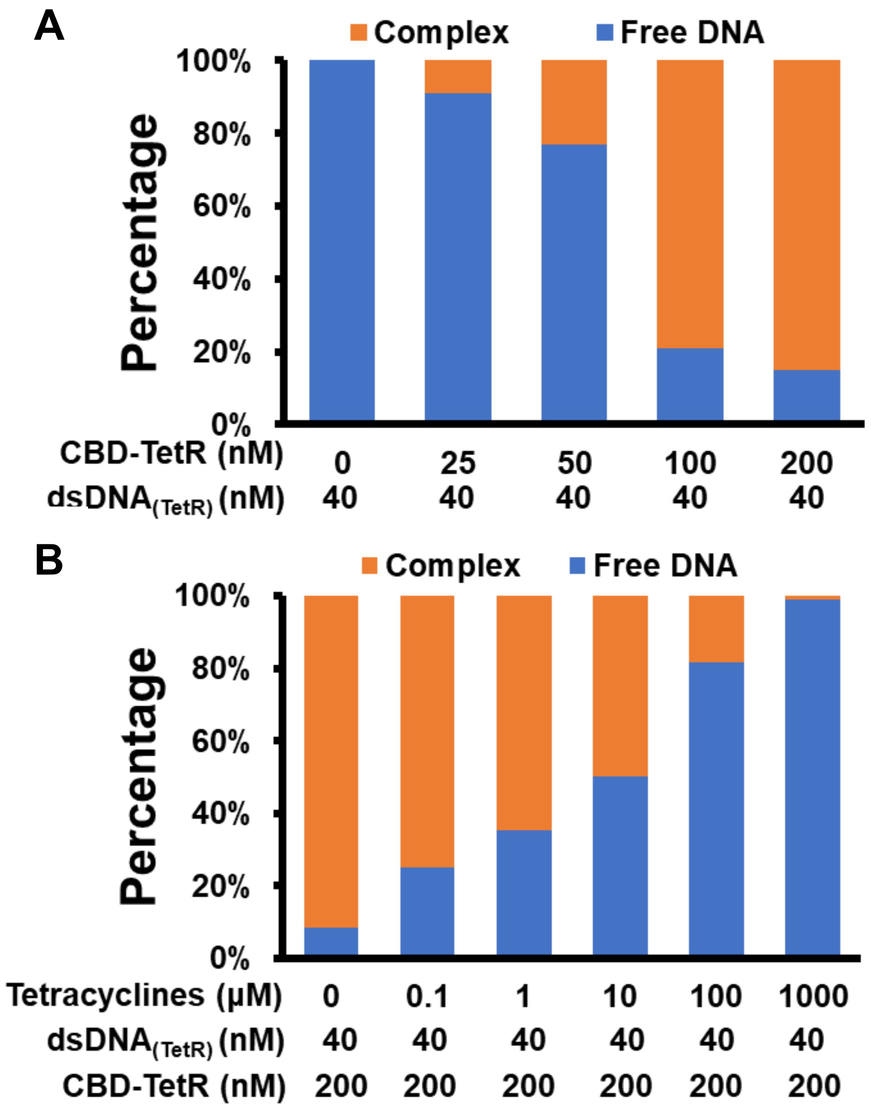

Supplementary Figure 4. Relatively quantitative analysis of EMSA data obtained by densitometric analysis of the dsDNA-CBD-TetR complex. **A.** EMSA with the CBD-TetR and dsDNA which contains the TetR binding motif. The concentration of $dsDNA_{(TetR)}$ was 40 nM, CBD-TetR was added to the system with a final concentration of 0, 25, 50, 100, 200nM. **B.** Evaluation of CBD-TetR allosteric activity in the presence of tetracyclines by EMSA. The concentrations of $dsDNA_{(TetR)}$ and CBD-TetR was 40nM and 200 nM, respectively. Tetracyclines was added to the system with a final concentration of 0, 0.1, 1, 10, 100 and 1000μM. Densitometric analysis was performed by gel image software (BioRad Inc.).

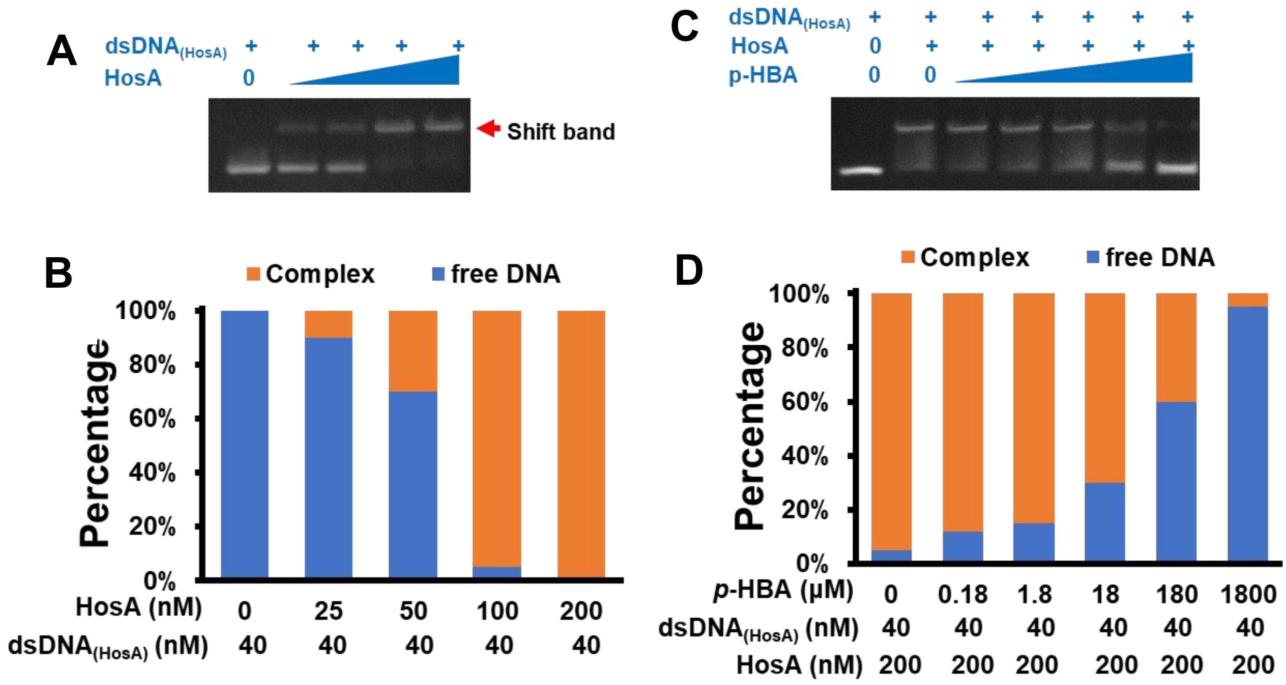

Supplementary Figure 5. Evaluation of dsDNA binding and allosteric activity of HosA by EMSA. **A.** EMSA with HosA and dsDNA which contains the HosA binding motif. The concentration of $dsDNA_{(HosA)}$ was 40 nM, HosA was added to the system with a final concentration of 0, 25, 50, 100, 200nM. **B.** Relatively quantitative analysis of EMSA with HosA and $dsDNA_{(HosA)}$. **C.** EMSA with HosA and $dsDNA_{(HosA)}$ in the presence of *p*-HBA. The concentrations of $dsDNA_{(HosA)}$ and HosA was 40nM and 200 nM, respectively. *p*-HBA was added to the system with a final concentration of 0, 0.18, 1.8, 18, 180 and 1800μM. **D.** Relatively quantitative analysis of dsDNA-HosA complex in the presence of *p*-HBA in EMSA. Densitometric analysis was performed by gel image software (BioRad Inc.).

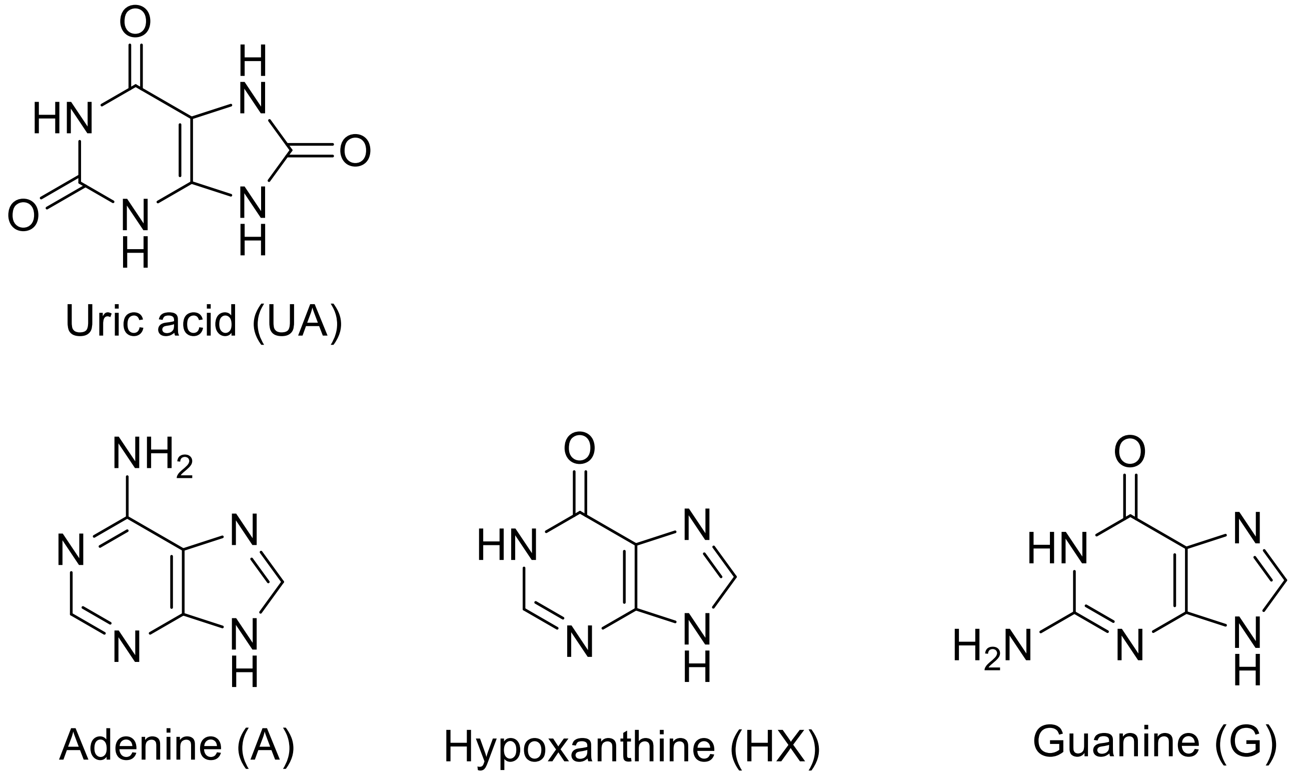

Supplementary Figure 6. Chemical structure of uric acid (UA), adenine (A), hypoxanthine (HX) and guanine (G).

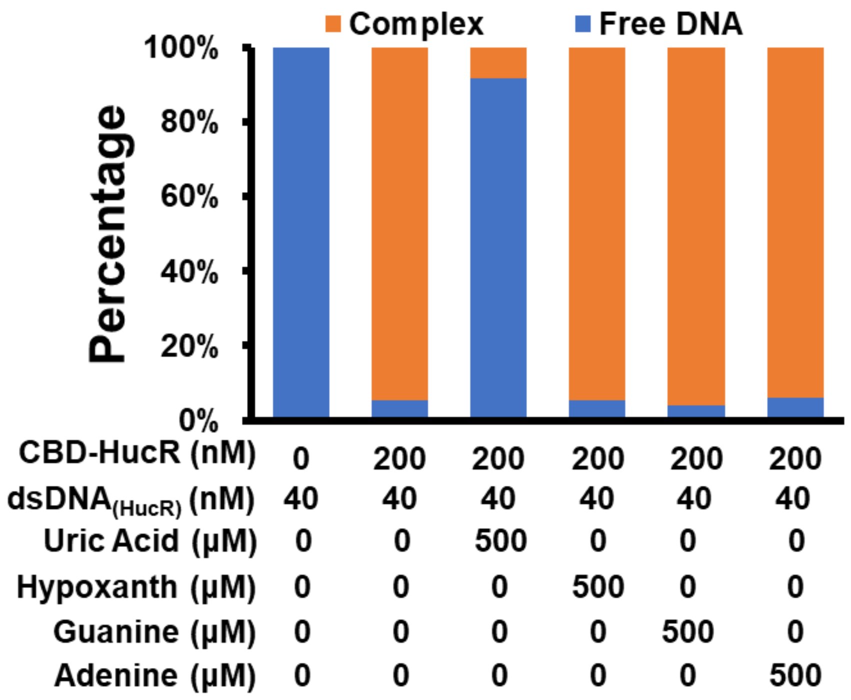

Supplementary Figure 7. Relatively quantitative analysis of dissociation of dsDNA form the CBD-HucR-dsDNA$_{(HucR)}$ complex in the presence of uric acid and its structure analogs. The final concentration of uric acid (UA), hypoxanthine (HX), guanine (G), adenine (A) in each reaction was 500 μM. The concentrations of dsDNA$_{(HucR)}$ and CBD-HucR was 40nM and 200 nM, respectively. Densitometric analysis was performed by gel image software (BioRad Inc.).

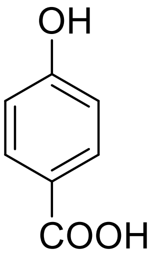

p-hydroxybenzoic acid
(*p*-HBA)

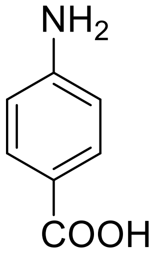

p-aminobenzoic acid
(p-ABA*)*

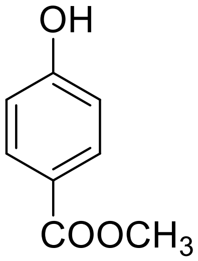

methyl-*p*-hydroxybenzoate
(*p*-MHB)

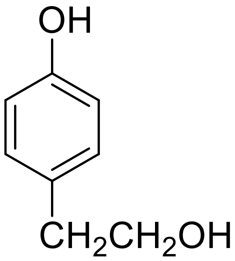

tyrosol

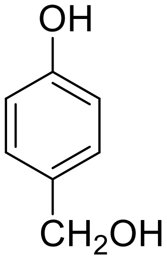

p-hydroxybenzyl alcohol
(*p*-HBnOH)

Supplementary Figure 8. Chemical structure of *p*-hydroxybenzoic acid (*p*-HBA), *p*-aminobenzoic acid (*p*-ABA), methyl-*p*-hydroxybenzoate (*p*-MHB), tyrosol and *p*-hydroxybenzyl alcohol (*p*-HBnOH).

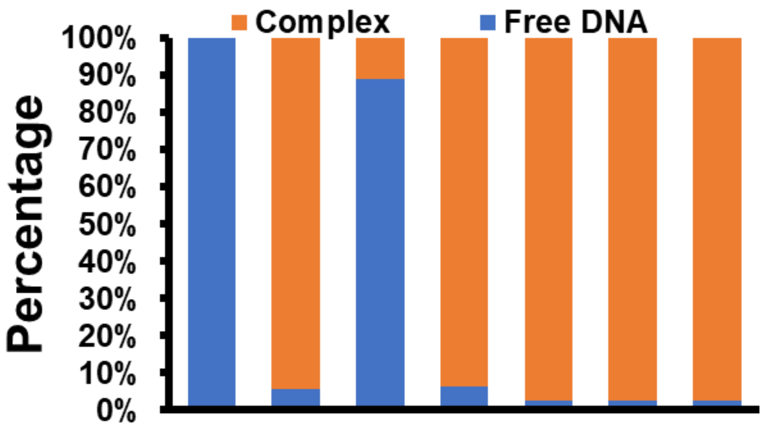

CBD-HosA (nM)	0	200	200	200	200	200	200
$dsDNA_{(HosA)}$ (nM)	40	40	40	40	40	40	40
p-HBA (mM)	0	0	1.8	0	0	0	0
p-HBnOH (mM)	0	0	0	1.8	0	0	0
p-MHB (mM)	0	0	0	0	1.8	0	0
p-ABA (mM)	0	0	0	0	0	1.8	0
Tyrosol (mM)	0	0	0	0	0	0	1.8

Supplementary Figure 9. Relatively quantitative analysis of dissociation of $dsDNA_{(HosA)}$ form the CBD-HosA-$dsDNA_{(HosA)}$ complex in the presence of p-hydroxybenzoic acid and its structure analogs. The final concentration of p-hydroxybenzoic acid (p-HBA), p-hydroxybenzyl alcohol (p-HBnOH), methyl-p-hydroxybenzoate (p-MHB), p-aminobenzoic acid (p-ABA) and tyrosol in each reaction was 1.8 mM. The concentrations of $dsDNA_{(HosA)}$ and CBD-HosA was 40nM and 200 nM, respectively. Densitometric analysis was performed by gel image software (BioRad Inc.).

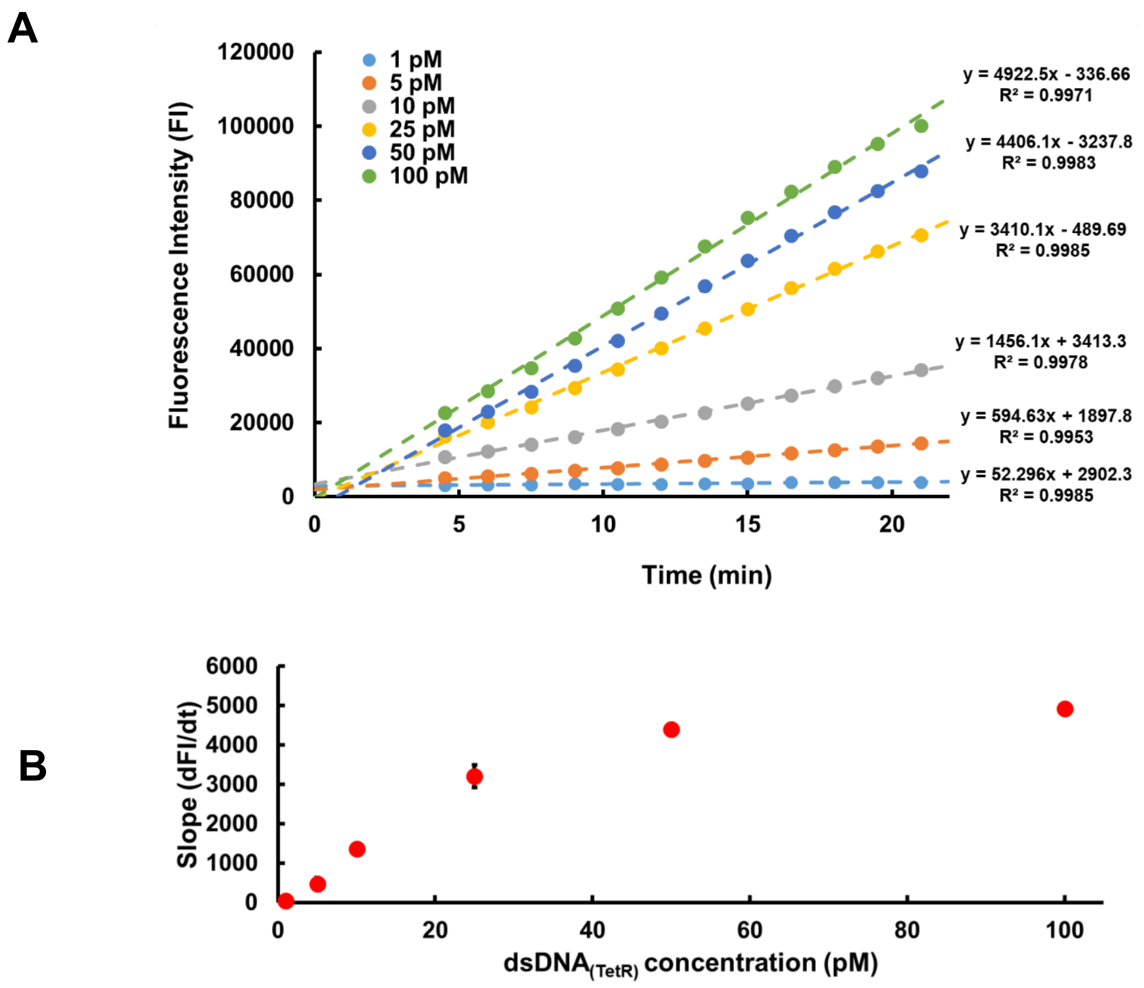

Supplementary Figure 10. Effects of different concentration of activator dsDNA(tetR) on the on the ssDNA trans-cleavage activity of CRISPR/Cas12a. In this figure, $dsDNA_{(tetR)}$ which contains TetR binding motif was used to active the *trans*-cleavage activity of CRISPR/Cas12a. **A.** The linear trend lines of fluorescence signal induced by different concentration of $dsDNA_{(tetR)}$. **B.** Response between slope of fluorescence intensity and $dsDNA_{(tetR)}$ concentration.

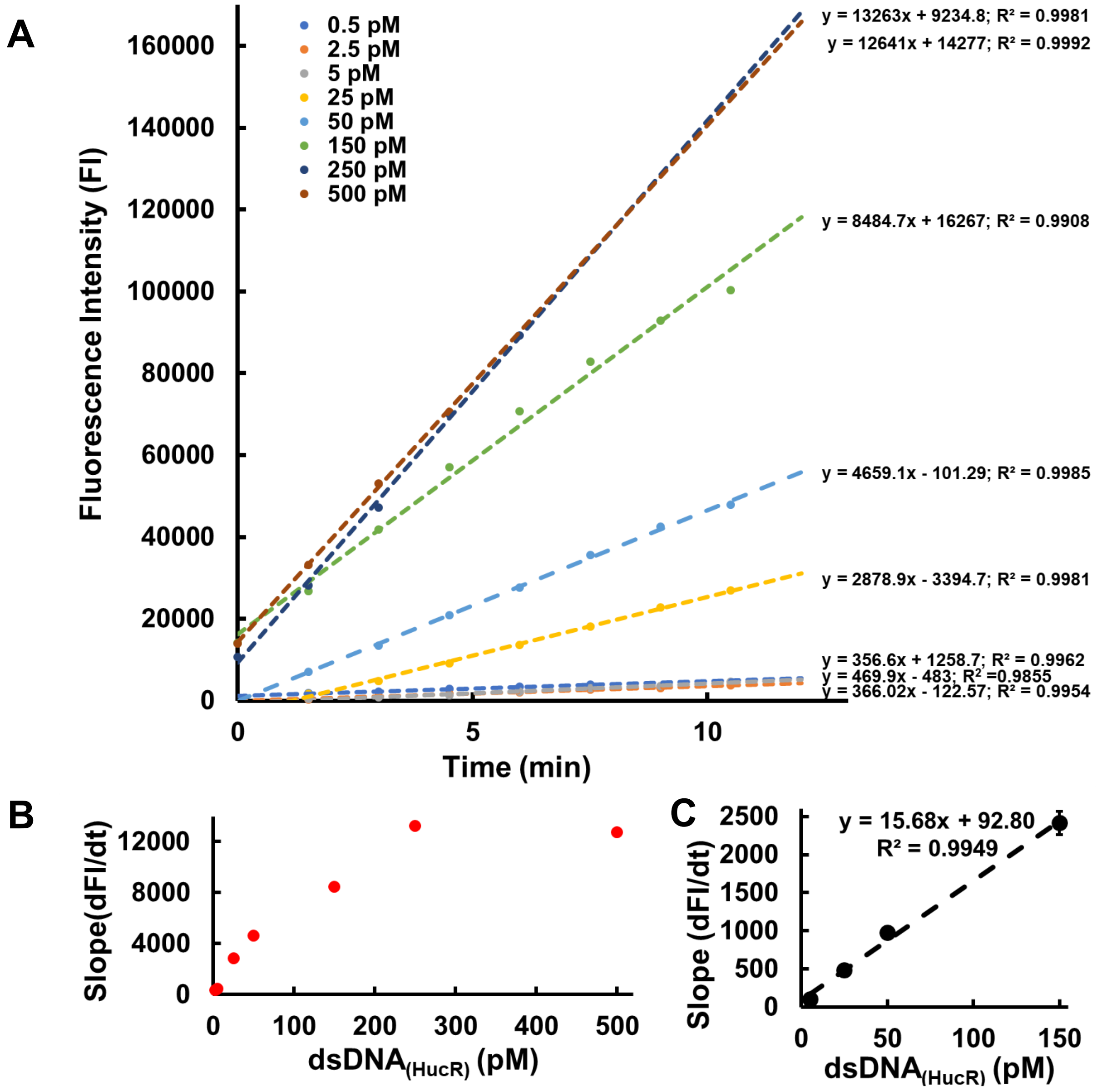

Supplementary Figure 11. Effects of different concentration of activator $dsDNA_{(HucR)}$ on the ssDNA trans-cleavage activity of CRISPR/Cas12a. **A.** The linear trend lines of fluorescence signal induced by different concentration of $dsDNA_{(HucR)}$. **B.** Response between slope of fluorescence intensity and $dsDNA_{(HucR)}$ concentration. **C.** The linear relationship between the concentration of $dsDNA_{(HucR)}$ and fluorescence intensity. Linearity range of the calibration curve was 5 to 150 pM (R^2 = 0.995). Error bars are means and SDs from at least two independent repeats.

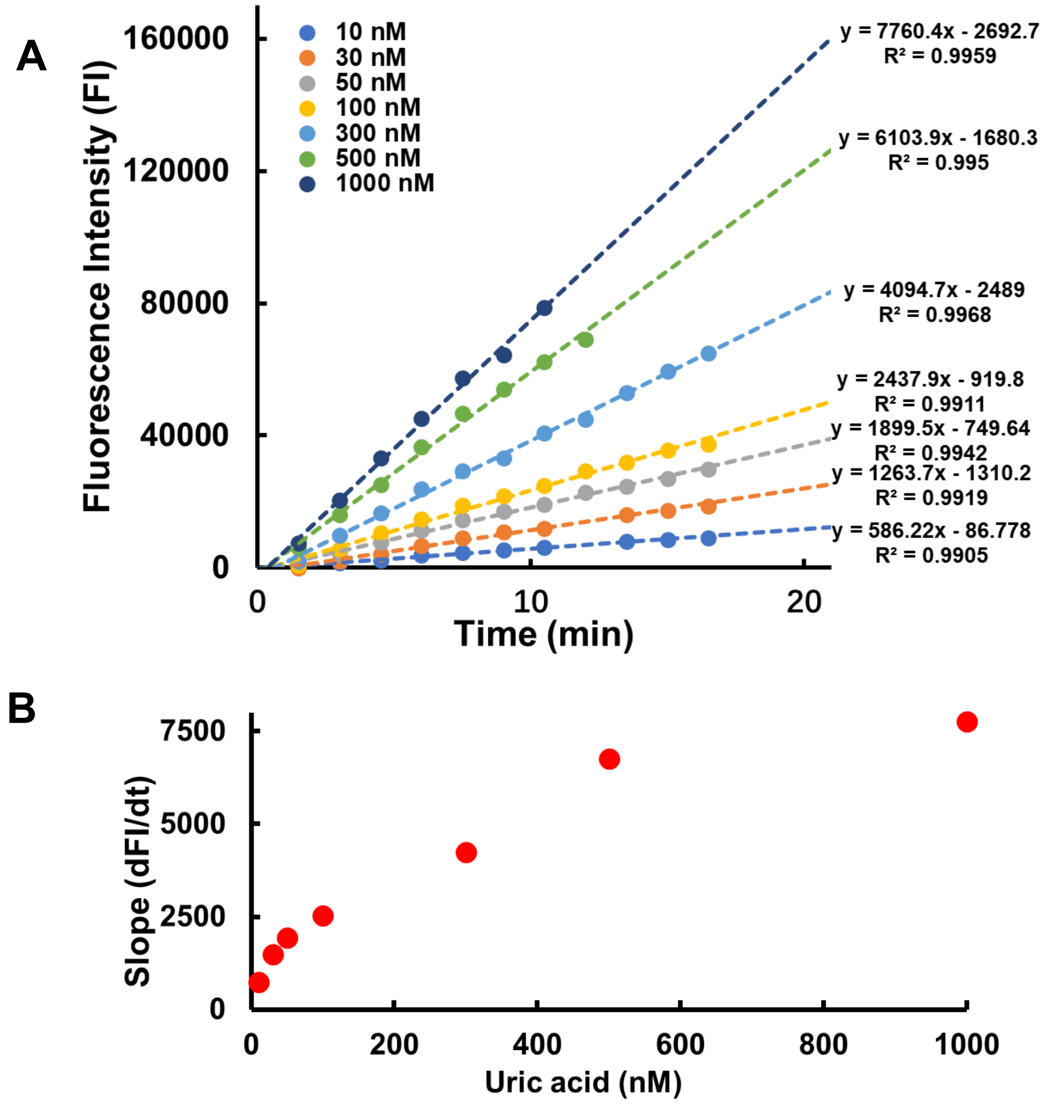

Supplementary Figure 12. Analysis of different concentration of uric acid by CaT-SMelor. **A.** The linear trend lines of fluorescence signal induced by different concentration of uric acid. **B.** Response between slope of fluorescence intensity and uric acid concentration.

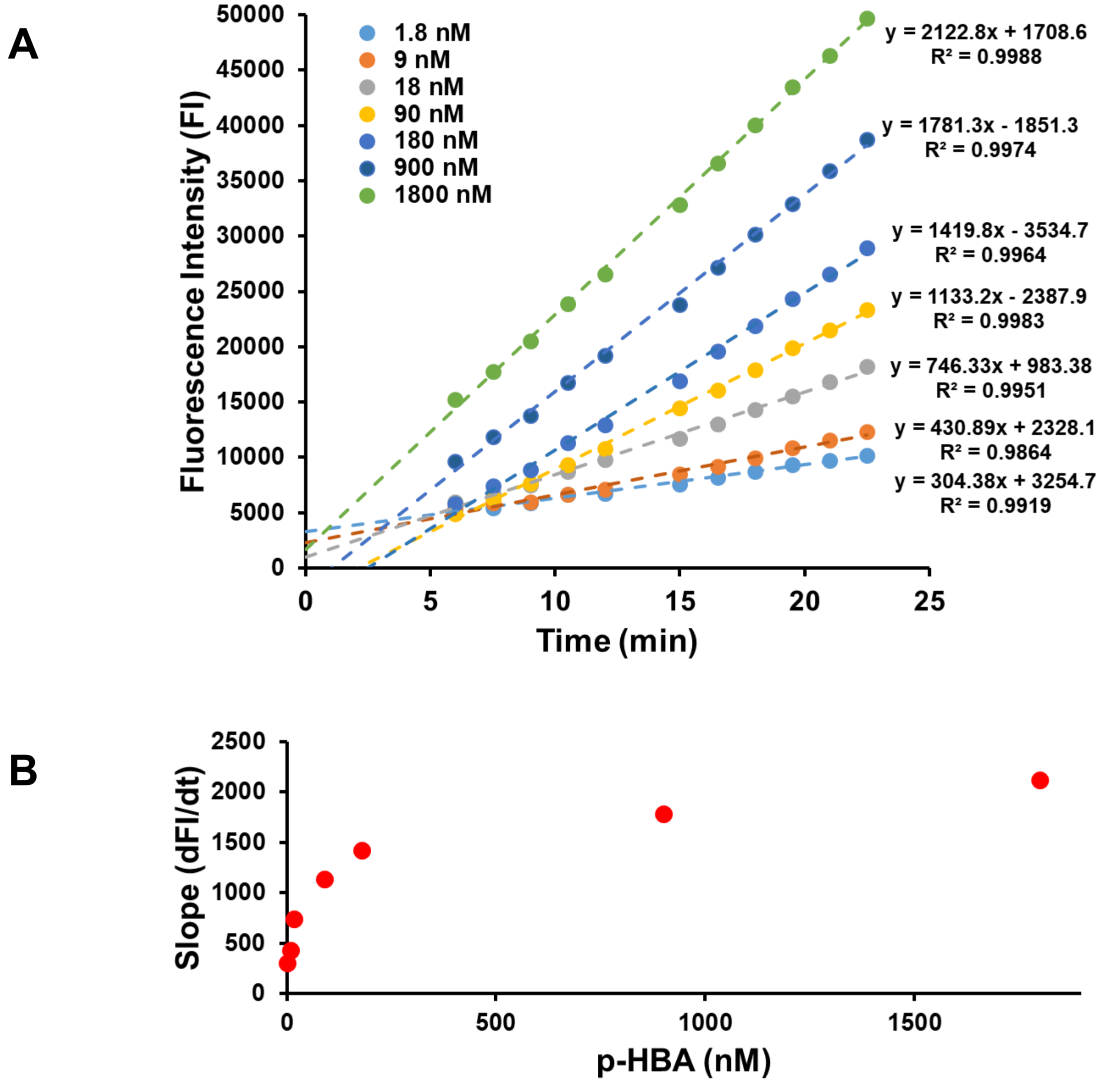

Supplementary Figure 13. Analysis of different concentration of p-hydroxybenzoic acid (*p*-HBA) by CaT-SMelor. **A.** The linear trend lines of fluorescence signal induced by different concentration of *p*-HBA. **B.** Response between slope of fluorescence intensity and *p*-HBA concentration.

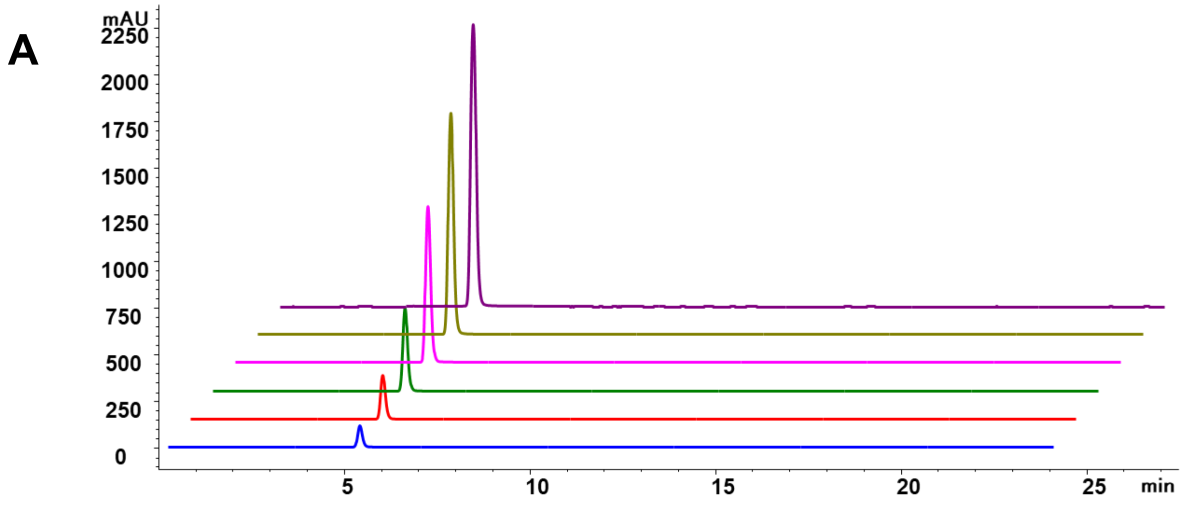

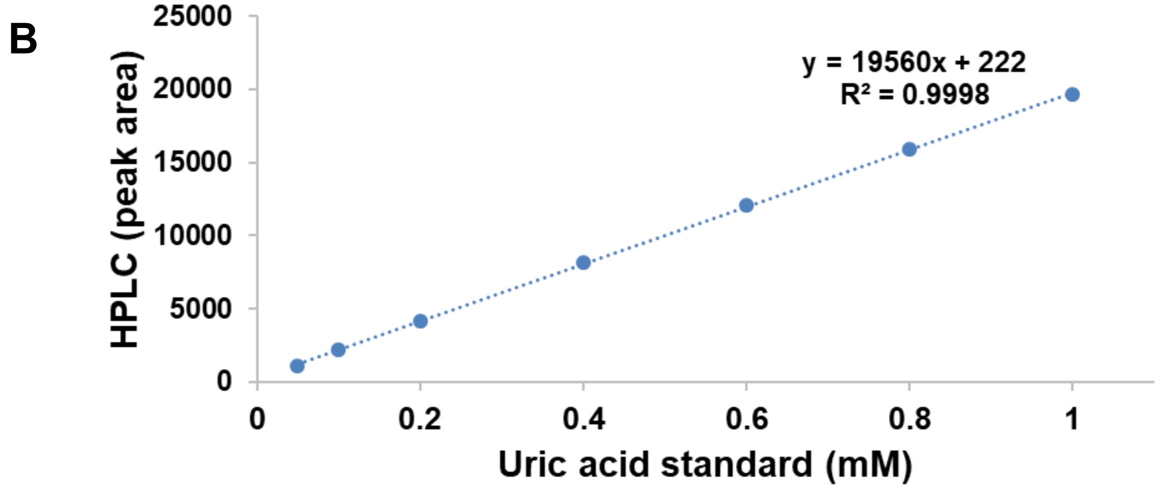

Supplementary Figure 14. Analysis of uric acid by high performance liquid chromatography (HPLC). **A.** HPLC profile of uric acid standard. **B.** HPLC standard curve of uric acid.

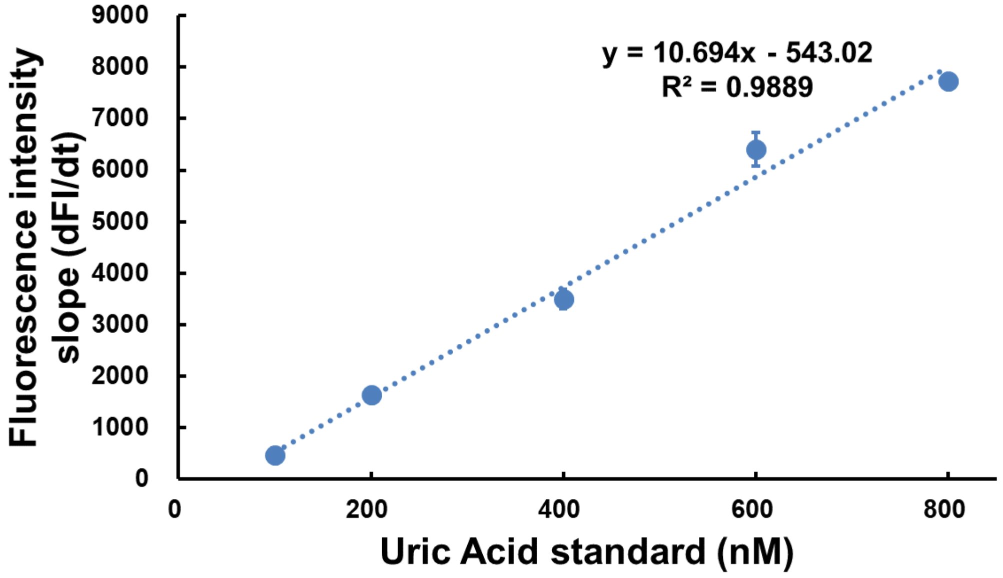

Supplementary Figure 15. CaT-SMelor standard curve of uric acid. Error bars are means and SDs from at least two independent repeats.

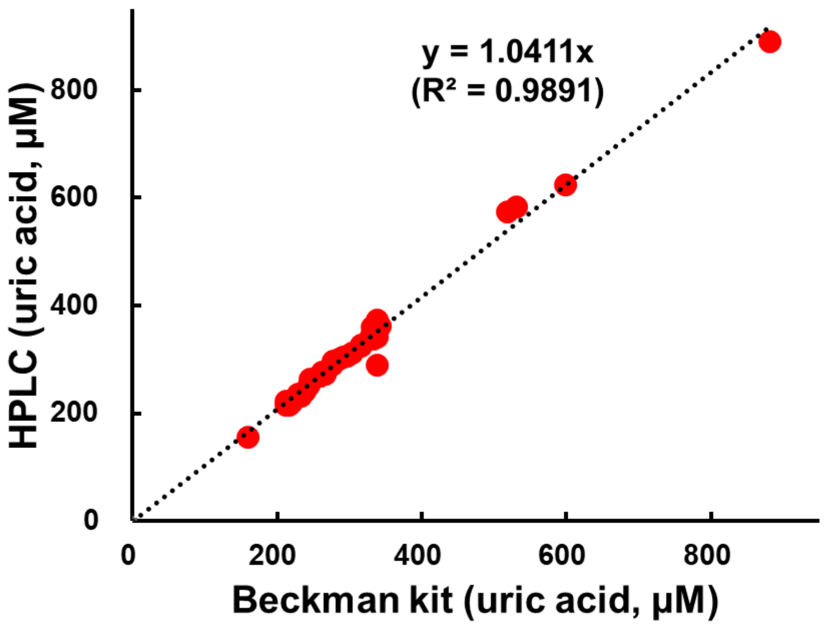

Supplementary Figure 16. Analysis of uric acid with a clinical automatic biochemical analyzer system and HPLC.

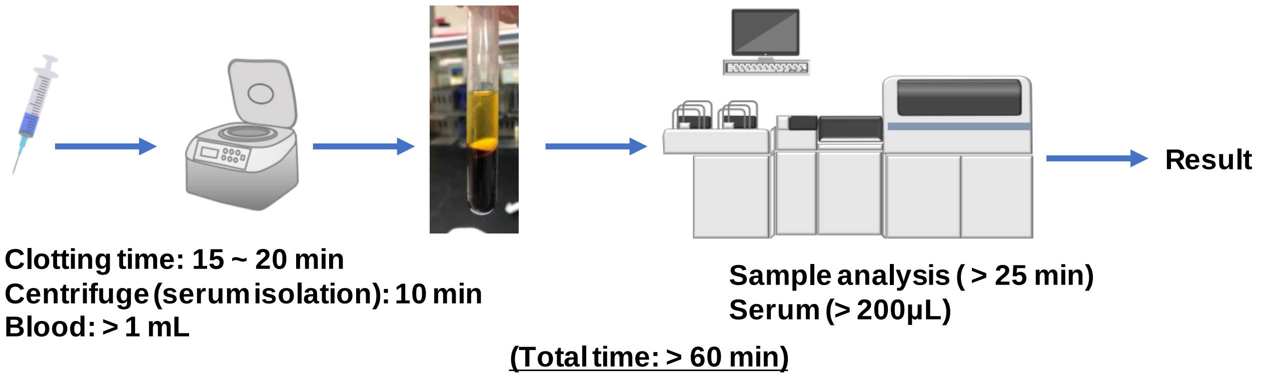

Supplementary Figure 17. Clinical laboratory analysis of blood by automatic biochemical analyzer system.

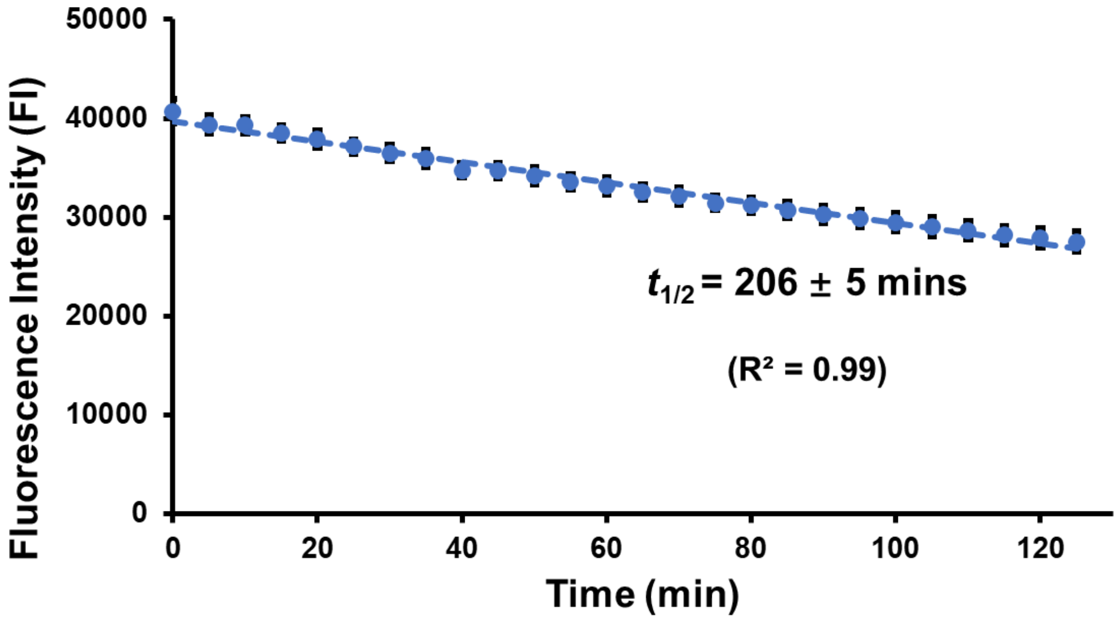

Supplementary Figure 18. Half-time of fluorescence signal. Based on the fluorescence attenuation curves (R^2 = 0.99), under present experiment condition, it was estimated that half-time of fluorescence signal was 206 ± 5 mins. Error bars are means and SDs from at least two independent repeats.

Supplementary Tables

Supplementary Table 1. Analysis of uric acid in blood samples with HPLC and CaT-SMelor.

Blood sample	CaT-SMelor (μM)	HPLC (μM)
B1	520 ± 13.3	508 ± 1.5
B2	570 ± 16.5	551 ± 0.7
B3	298 ± 17.1	280 ± 0.7
B4	262 ± 15.3	242 ± 0.3
B5	200 ± 19.2	192 ± 0.3

Values are mean± SD of two replications.

Supplementary Table 2. Cost accounting of CaT-SMelor reaction system.

Items	Consumption and cost (per 20 μL reaction)	Price & Sources
Cas12a	1 pmol; ~ 0.13$	2000 pmol, 250$, NEB
RNase Inhibitor	4 U; ~ 0.04$	25000 U, 250$, Takara
aTF	10 pmol; ~ 0.01$	N/A
MC	100 μg; < 0.01$	100g, 15$, Sangon Biotech
dsDNA	1pmol; < 0.01$	5 nmol, 15$, Genscript
crRNA	1pmol; ~ 0.02$	5 nmol, 100$, Genscript
Probe	5 pmol; < 0.05$	5 nmol, 45$, Genscript

MC: Microcrystalline Cellulose; aTF: allosteric transcription factor; dsDNA: double strand DNA.

Supplementary Table 3. The strains and plasmid used in this study

Strains and plasmids	Features	Sources
E.coli DH10B	F– *mcrA* *Δ*(*mrr-hsdRMS-mcrBC*) ϕ80*dlacZΔM15* *ΔlacX74 deoR recA1 endA1 araΔ139* *D*(*ara leu*)*1697 galU galK λ*– *rpsL nupG*	GIBCO-BRL
E. coli BL21(DE3)	F- *ompT hsdSB(rB- mB-) gal dcm* （DE3）	TIANGEN, Shangai
pET35b	Kan, P_{T7}, CBD	This study
pET28a	kan, P_{T7}, His-tag	Novagen
pET28TEV	LbCas12a in pET28a	Tolobio, Shanghai
pECUST- ZL0031	*hosA*-CBD* in pET28a	This study
pECUST- ZL0032	*hucR*-CBD** in pET28a	This study
pECUST- ZL0033	*tetR*-CBD*** in pET28a	This study
pECUST- ZL0034	*tetR* in pET28a	This study
pECUST- ZL0035	*hucR* in pET28a	This study
pECUST- ZL0036	*hosA* in pET28a	This study

* Amino acid sequence of CBD-HosA:

MIAADSLPGVYMALRNKAFHQLRQLFQQHTARWQHELPDLTKPQYAVMRAIADKPGIEQV
ALIEAAVSTKATLAEMLARMENRGLVRREHDAADKRRRFVWLTAEGEKVLAAAIPIGDSVDA
EFLGRLSGEEQELFMQLVRKMMSKLESGGGGSGGGGSGGGGMSVEFYNSNKSAQTNSI
TPIIKITNTSDSDLNLNDVKVRYYYTSDGTQGQTFWCDHAGALLGNSYVDNTSKVTANFVK
ETASPTSTYDTYVEFGFASGAATLKKGQFITIQGRITKSDWSNYTQTNDYSFDASSSTPVVN
PKVTGYIGGAKVLGTAPLEHHHHHH

** Amino acid sequence of CBD-HucR:

MSARMDNDTAALLERIRSDWARLNHGQGPDSDGLTPSAGPMLTLLLLERLHAALGREIERT
YAASGLNAAGWDLLLTLYRSAPPEGLRPTELSALAAISGPSTSNRIVRLLEKGLIERREDER
DRRSASIRLTPQGRALVTHLLPAHLATTQRVLAPLSAQEQRTLEELAGRMLAGLEQGVLES

GGGGSGGGGSGGGGMSVEFYNSNKSAQTNSITPIIKITNTSDSDLNLNDVKVRYYYTSDG
TQGQTFWCDHAGALLGNSYVDNTSKVTANFVKETASPTSTYDTYVEFGFASGAATLKKGQ
FITIQGRITKSDWSNYTQTNDYSFDASSSTPVVNPKVTGYIGGAKVLGTAPLEHHHHHH

*** Amino acid sequence of CBD-TetR：

MSRLDKSKVINSALELLNEVGIEGLTTRKLAQKLGVEQPTLYWHVKNKRALLDALAIEMLDR
HHTHFCPLEGESWQDFLRNNAKSFRCALLSHRDGAKVHLGTRPTEKQYETLENQLAFLC
QQGFSLENALYALSAVGHFTLGCVLEDQEHQVAKEERETPTTDSMPPLLRQAIELFDHQGA
EPAFLFGLELIICGLEKQLKCESGSLESGGGGSGGGGSGGGGMSVEFYNSNKSAQTNSIT
PIIKITNTSDSDLNLNDVKVRYYYTSDGTQGQTFWCDHAGALLGNSYVDNTSKVTANFVKE
TASPTSTYDTYVEFGFASGAATLKKGQFITIQGRITKSDWSNYTQTNDYSFDASSSTPVVNP
KVTGYIGGAKVLGTAPLEHHHHHH

Supplementary Table 4. The sequence of PCR primers for plasmid construction

Primer	Sequence (5' - 3')
hucR-CBD-F	gtggtggtggtggtgctcgagtggtgctgtaccaagaactt
hucR-CBD-R	tgtgaaagtgggtctctcgagtctggcggcggcggctctggcggcggcggctctggcggcg gcggcatgtcagttgaattttaca
hosA-CBD-R	aagatgatgagcaaactcgagtctggcggcggcggctctggcggcggcggctctggcggc ggcggcatgtcagttgaattttaca
tetR-F	ctcgagagacccactttcac
tetR-R	aagaaggagatatacatatgtctagattagataaaag
tetR-CBD-R	tgtgaaagtgggtctctcgagtctggcggcggcggctctggcggcggcggctctggcggcg gcggcatgtcagttgaattttaca

Supplementary Table 5. The sequence of crRNA, dsDNA activator and ssDNA reporter etc.

Name	Sequence (5' - 3')
crRNA	
T7-promoter-F	GAAATTAATACGACTCACTATAGGG
crRNA-hucR-R	TAGGTAGACATCTAAGTAATCTACAACAGTAGAAATTCCCTATAGTGAGTCGTATTAATTTC
crRNA-tetR-R	CTCTATCACTGATAGGGAATCTACAACAGTAGAAATTCCCTATAGTGAGTCGTATTAAT
crRNA-HosA-R	ACTGTTCGTATACGAACGATCTACAACAGTAGAAATTCCCTATAGTGAGTCGTATTAATTTC
dsDNA	
dsDNA$_{(HucR)}$-F	tactgagccatgtatccaggtcattgTACTTAGATGTCTACCTAagctctgacagttcca
dsDNA$_{(HucR)}$-R	tggaactgtcagagctTAGGTAGACATCTAAGTAcaatgacctggatacatggctcagta
dsDNA$_{(Tet\mathit{R})}$-F	tgagccatgtatccaggtcatttgtccctatcagtgatagagaagctctgacagttcca
dsDNA$_{(Tet\mathit{R})}$-R	tggaactgtcagagcttctctatcactgatagggacaaatgacctggatacatggctca
dsDNA$_{(HosA)}$-F	tactgagccatgtatccaggtcattgCGTTCGTATACGAACAgtagctctgacagttcca
dsDNA$_{(HosA)}$-R	tggaactgtcagagctacTGTTCGTATACGAACGcaatgacctggatacatggctcagta
ssDNA	
Reporter*	gattagcgtacgcacgttac

*5' labeled with 5-FAM, 3' labeled with BHQ.
